# A Critical Review of Published Data on the Gas Temperature and the Electron Density in the Electrolyte Cathode Atmospheric Glow Discharges

**DOI:** 10.3390/s120506576

**Published:** 2012-05-18

**Authors:** Pál Mezei, Tamás Cserfalvi

**Affiliations:** 1 Institute for Solid State Physics and Optics, Wigner Research Centre for Physics, Hungarian Academy of Sciences, H-1525 Budapest, P.O. Box 49, Hungary; 2 Aqua-Concorde Water Analysis R&D LLC, T. Meisel Laboratory, Budapest, Hungary; E-Mail: cserfalvi@yahoo.com

**Keywords:** direct multielement sensor, metals in water, atmospheric glow plasma

## Abstract

Electrolyte Cathode Discharge (ELCAD) spectrometry, a novel sensitive multielement direct analytical method for metal traces in aqueous solutions, was introduced in 1993 as a new sensing principle. Since then several works have tried to develop an operational mechanism for this exotic atmospheric glow plasma technique, however these attempts cannot be combined into a valid model description. In this review we summarize the conceptual and technical problems we found in this upcoming research field of direct sensors. The T_G_ gas temperature and the n_e_ electron density values published up to now for ELCAD are very confusing. These data were evaluated by three conditions. The first is the gas composition of the ELCAD plasma, since T_G_ was determined from the emitted intensity of the N_2_ and OH bands. Secondly, since the ELCAD is an atmospheric glow discharge, thus, the obtained T_G_ has to be close to the T_e_ electron temperature. This can be used for the mutual validation of the received temperature data. Thirdly, as a consequence of the second condition, the values of T_G_ and n_e_ have to agree with the Engel-Brown approximation of the Saha-equation related to weakly ionized glow discharge plasmas. Application of non-adequate experimental methods and theoretical treatment leads to unreliable descriptions which cannot be used to optimize the detector performance.

## 1. Introduction

**Figure 1.**
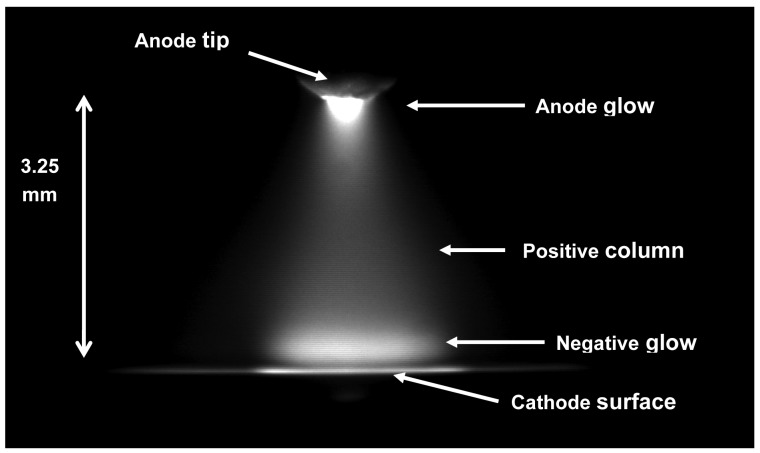
The CCD camera picture of the typical ELCAD plasma operating between the electrolyte cathode and the W anode [4].

The electrolyte cathode atmospheric glow discharge (ELCAD) technique was invented for the direct measurement of metals (Zn, Cd, Cu, Ni, Cr, Pb, alkali and earth metals, Fe, Mn, *etc.*) in aqueous samples [1]. In the case of ELCAD, the sample solution is the cathode and a W-rod above it (3–5 mm) is the anode. Under atmospheric air pressure, a d.c. glow discharge is produced (Figure 1). The atomic lines of metals dissolved in the solution appear in the spectrum emitted by the ELCAD, and in this way the concentration of metals in a sample can be determined. Since the cathode sputtering consumes the sample solution, the maintenance of a constant electrode distance, thus a stable discharge operation requires a constant flow rate of cathode solution [1].

The atomic emission having very narrow emitted lines provides excellent possibilities for the simultaneous multimetal detection of up to 20–30 elements. In a flow injection analytical system, the capillary ELCAD detector reached approximately 1 ng mass detection limits (14–34 ng/mL) for heavy metals [2]. Later, in a continuous-flow method, the limits of detection are reported to be between 0.8–350 ng/mL for 16 metals ranging from Na to Hg [3].

The emitted intensity of the atomic lines of metals dissolved in the cathode solution, which have a maximum in the negative glow region, are determined by the pressure, the current and the solution pH, hence fall also on the cathode [4–6]. This was explained by the fact that the M^+^ positive metal ions leave the cathode solution due to the cathode sputtering. In the cathode dark space, these M^+^ ions are recombined by the reaction M^+^ + 2e → M + e. The rate of this recombination is inversely proportional to the kT_e_ average electron energy [7]. The produced neutral M metal atoms diffuse into the negative glow, where they are mainly excited by electron impact [4–6].

On the other hand, the T_G_ gas temperature and the n_e_ electron density also influence the emitted intensity of the atomic metal lines. T_G_ relates to the gas particle density, the collision number between the electrons and the gas particles, the mean free path of electrons and hence the electron energy gained in this distance [8–10]. In this way, the T_G_ and the n_e_ are two basic parameters of the ELCAD plasma determining the operation and the excitation mechanisms, hence the emitted intensities as well.

In the case of the ELCAD and its homologue plasmas, the T_G_ was determined mainly by means of the emitted band of N_2_ molecule and OH radical, while the n_e_ was studied by various methods. The published data, however, are very confusing, covering ∼3 orders of magnitude in n_e_ values and ∼1 order of magnitude in T_G_ values [3,4,6,11–20,21].

The evaluation of the available data was based on the following three conditions:

1. In order to offer an accurate method for the determination of T_G_, the gas composition of the ELCAD plasma was studied.
2. Since the ELCAD is an atmospheric glow discharge, hence for T_G_ and T_e_ electron temperature, T_G_ = T_e_ [22,23] or T_e_ ≈ T_G_ [24–26] can be expected. If T_e_ is also measured, T_e_ = T_G_ or T_e_ ≈ T_G_ relation can be used for the mutual validation of the received values.
3. As a consequence of the T_e_ = T_G_ (or T_e_ ≈ T_G_) relation referring to an local thermodynamic equilibrium (LTE) [22,23] (or a good approximation of it [24–26]), the corresponding values of T_G_ and n_e_ can be calculated from the Engel-Brown approximation [8,10] of the Saha-equation [27,28] related to the weakly ionized glow discharges with low charge densities.

## 2. The Published T_G_ and n_e_ DATA

The data presented in Table 1 are evaluated by means of the three conditions mentioned above.

## 3. The Evaluation of the Published T_G_ and n_e_ Data

### 3.1. The Investigation of the Gas Composition

To obtain the correct T_G_ in the ELCAD plasma, the first necessary condition is the accurate knowledge of gas composition of the ELCAD plasma. From the measurement of the minimum flow rate of the electrolyte cathode, which can still sustain the discharge for at least 10 s, a cathode sputtering rate of 1,500 mg/min was obtained at a cathodic current density of 3.7 A/cm^2^, a current of 80 mA, and a pH = 1.55 (adjusted with HCl). This means, that 5 × 10^22^ H_2_O molecules leave the electrolyte cathode each minute due to the cathode sputtering. After the ELCAD plasma is ignited in the atmospheric air, the plasma composition is changing very fast by the cathode sputtering.

**Figure 2.**
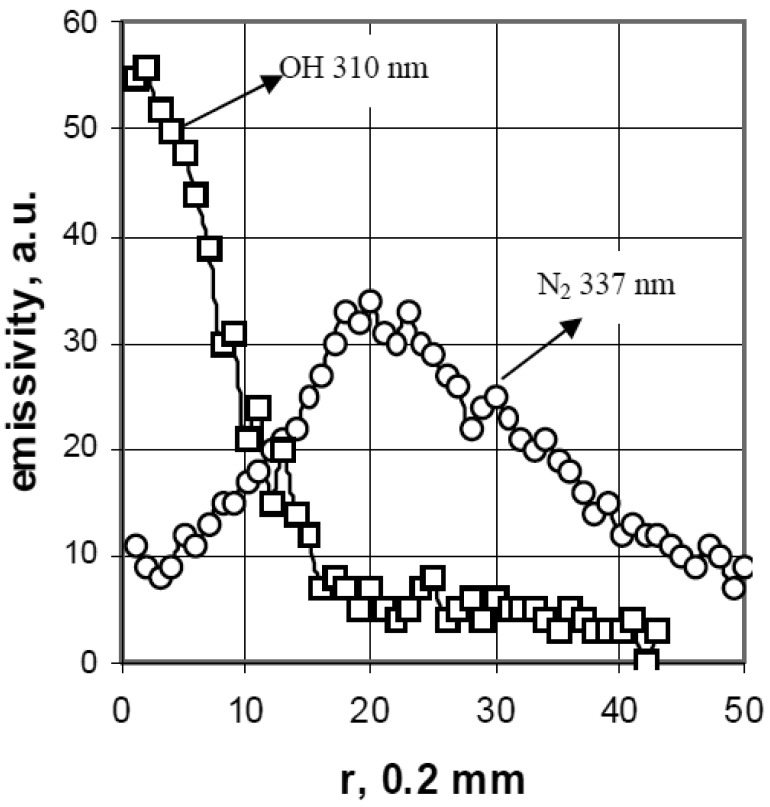
The radial distribution of the emitted intensity of OH 310 nm (squares) and the N_2_ 337 nm (circles) in the negative glow region of a typical ELCAD discharge (I = 67 mA, tap water, pH = 1.55) [31].

This sputtering rate is higher by 3–4 orders of magnitude than those observed on metal cathodes. Since this high flux of the sputtered matter must leave the discharge plasma through its boundary surface, an overpressure builds up in the core of the plasma. Due to this overpressure, the solution cathode surface is depressed [29] and a pressure gradient appears between the plasma core and the outer, ambient air. Therefore, a significant gas flow from the plasma core to the ambient air occurs. In ELCAD, the T_G_ values of ∼8,000–5,000 K were found from the ratio of the measured intensity of the OH 306.5 nm, 306.8 nm and 308.9 nm unresolved band heads [6], hence the thermal water splitting effect appears producing H and OH particles [30]. This multiplies further the rate of outward gas flow. Thus, the OH radicals produced in the plasma core leave the core of the cathode dark space and the negative glow with a velocity of 5–10 m/s. This totally obstructs the diffusion of any component of the ambient gas atmosphere into the ELCAD plasma [31]. Because of this extensive flushing process the ELCAD plasma operates in a self-generated saturated water vapor internal atmosphere. This is supported by the measured intensity distributions:

The intensity distribution of the OH-310 nm and the N_2_-337 nm bands in the ELCAD measured by an ultraviolet sensitive CCD camera using the corresponding interference filters (λ_0_ = 310 nm, λ_0_ = 337 nm, Δλ = 10 nm). The Abel-inversion processing of these plasma pictures show that in the near cathode region, the plasma contains dominantly OH radicals, while N_2_ can be observed only in the outer sheath of the plasma (Figure 2 [31]).

The axial intensity distribution of N_2_ measured as a function of the distance from the anode showed only a peak in the close anode region, while it was very low at the other segment of the discharge. N_2_ can diffuse into the ELCAD plasma only at the near-anode region, since there the outflow of the plasma gases is practically negligible [6].

Since the ELCAD operates in a self-generated saturated water vapour with an atmospheric pressure, therefore the intensity of the O^+^ (441–445 nm) lines, the H_β_-486.1 nm line, the OH ultraviolet bands and the atomic lines of metals dissolved in the liquid electrolyte cathode were found to be independent from the applied outer gas atmosphere [4–6]. Considering these facts, the correct T_G_ values in the ELCAD plasma can be determined only from the emitted intensity of the OH bands.

Furthermore, the T_rot_ rotational temperature of the ultraviolet OH (A^2^Σ, v = 0) → OH (X^2^∏, v = 0) band was found to be close to the T_G_ [32], and Izarra demonstrated that this T_rot_ can be obtained from the measured intensity ratio of the G_0_ = 306.5 nm, the G_1_ = 306.8 nm and the G_ref_ = 308.9 nm (G_0_/G_ref_; G_1_/G_ref_) unresolved band heads [33]. He gave the T_rot_ values in 100 K steps as a function of the spectral resolution of the applied monochromator. The received T_rot_ values were verified by an independent, interferometric measurement [34].

The result of other methods (the Boltzmann-plot, the simulation of emitted spectrum as a function of T_G_), applied for determination of T_G_ was not verified by an independent measurement [3,11–20]. Therefore, the Izarra method can be considered to be only a confirmed determination of the correct T_G_ value in the ELCAD plasma.

### 3.2. The T_e_ Electron and the T_G_ Gas Temperatures

Since the ELCAD is an atmospheric glow discharge, thus T_G_ ≈ T_e_ can be expected [22–26]. This is valid only for the data in the first line of Table 1. In this case, T_G_ is determined from the emitted intensity of the OH 306–309 nm bands with using of Izarra method, while T_e_ is obtained from the ratio of measured intensity of the Cu-I 510.5 nm and 515.3 nm lines [4,6].

In all other cases, even if the T_G_ determined from the emitted intensity of the OH bands, T_e_ ≫ T_G_ was obtained. This was attributed to that the discharge plasma is not in the local thermodynamical equilibrium, but this explanation is a self-contradiction, since the Maxwell-Boltzmann distribution was applied for determination of T_rot_ by means of the Boltzmann-plot method and the simulation of the emitted spectrum of the OH bands.

The so called ionic temperature of T_ion_ ≈ 4,623–5,038 K was determined from the Saha-Eggert equation with using the measured intensity of the Mg-I 285.2 nm and the Mg-II 279.5 nm lines [3,16]:

(1/a) $$n_{e}\cdot \frac{I_{kl}^{+}\cdot \lambda_{kl}^{+}\cdot A_{pq}}{I_{pq}\cdot \lambda_{pq}\cdot A_{kl}^{+}}=\left(\frac{2g_{k}}{g_{p}}\right)\cdot {\left(\frac{2\pi \cdot m_{e}\cdot k\cdot T}{h^{2}}\right)}^{\overset{3}{\;}/\underset{2}{\;}}\cdot \exp\left(\frac{-(E_{i}+E_{k}-E_{p})}{k\cdot T}\right)$$

where *I*^+^*_kl_*, *λ*^+^*_kl_*, *A*^+^*_kl_* and *E_k_* are the measured intensity, the wavelength, the transition probability and the upper level energy of the Mg-II 279.5 nm transition. *I_pq_*, *λ_pq_*, *A_pq_* and *E_p_* are the same physical quantities corresponding to the Mg-I 285.2 nm transition. In this way, in the negative glow T_ion_ = (5,038 ± 60) K, and in the positive column T_ion_ = (4,623 ± 34) K values were obtained [3,16].

Equation (1/a) can be obtained from the Saha-Equation [27,28]

(1/b) $$\frac{n_{e}\cdot n_{i}}{n_{n}}=G\cdot {\left(\frac{2\pi \cdot m_{e}\cdot k\cdot T_{G}}{h^{2}}\right)}^{\overset{3}{\;}/\underset{2}{\;}}\cdot \exp\left(\frac{-e\cdot U_{i}}{k\cdot T_{G}}\right)$$

if the density of the neutral (*n_n_*) and the ionic (*n_i_*) particles is described by the Boltzmann distribution:

(2) $$n_{n}=n_{n0}\cdot \exp\left(-\frac{E_{p}}{kT_{e}}\right),n_{i}=n_{i0}\cdot \exp\left(-\frac{E_{k}}{kT_{e}}\right)$$

the emitted intensities are:

(3) $$I_{pq}=n_{n}A_{pq}hc/\lambda_{pq},I_{kl}^{+}=n_{i}A_{kl}^{+}hc/\lambda_{kl}^{+}$$

and:

(4) $$T_{G}=T_{e}$$

The conditions of Equations (2) and (4) can be applied for the ELCAD, since the excitations are mostly the electron impacts in it and it is an atmospheric glow discharge. Because of Equation (4), the received T_ion_ ≈ 4,623–5,038 K is not the so called ionic, but this is really the common temperature of T_G_ = T_e_. Therefore, the T_rot_ ≈ 3,200–3,600 K gas temperature determined from the emitted intensity of the OH 306 nm band is inaccurate.

### 3.3. T_G_ and n_e_ Values and the Engel-Brown Approximation

The Saha-equation concerns the highly ionized plasmas with high charge densities [27,28]. But the glow discharges are weakly ionized plasmas, hence their charge densities are very low. Since the ELCAD is also a glow discharge, hence, instead of Saha-equation, the Engel-Brown approximation given for glow discharges [8,10] is used for checking the published T_G_ and *n_e_* values:

(5) $$\frac{n_{e}}{n_{n}}∼\exp\left(\frac{-eU_{i}}{2\cdot k\cdot T_{G}}\right)$$

where *n_e_* and *n_n_* are the electron and neutral particle densities, *e* is the elementary charge, *k* is the Boltzmann constant and *U_i_* is the ionization potential of the gas.

The dependence of *n_n_* neutral gas particle density on the gas pressure and the gas temperature is [9]:

(6) $$n_{n}[{\text{cm}}^{-3}]=\frac{3.3\cdot 10^{16}\cdot p[\text{torr}]\cdot 298[K]}{T_{G}[K]}$$

Combining the Equations (5) and (6) and taking into account that the ELCAD operates in a saturated atmospheric pressure water vapour, thus *p* = 760 torr and *eU_i_* = 2 × 10^−18^ J, we have:

(7) $$n_{e}[{\text{cm}}^{-3}]∼\frac{7.35\cdot 10^{21}}{T_{G}}\cdot \exp\left(\frac{-2\cdot 10^{-18}}{2.76\cdot 10^{-23}\cdot T_{G}}\right)$$

The published T_G_ and *n_e_* values are compared with the results obtained from Equation (7). The *n_e_* ≈ 2.1 × 10^13^ cm^−3^ value was estimated [4,21] at the end of the cathode dark space, where *n_e_* ≈ *n*^+^(= positive ion density) [8–10]. To obtain the n_e_ value in the negative glow, this latter result needs a refinement due to the general charge density distributions.

For this saturated, atmospheric water vapour plasma, reliable simulated charge density distributions confirmed by experiments cannot be found in the literature. Therefore, the *n_e_* value in the negative glow can be estimated only on the base of two different general charge density distributions for glow discharges:

- The von Engel distribution indicates that *n_e_* in the negative glow is higher by a factor of ∼1.5 than that at the end of cathode dark space [10]. Thus:
  (8/a) $$n_{e}\approx 3\times 10^{13}{\text{cm}}^{-3}$$
- The Raizer distribution shows that n_e_ in the negative glow is lower by a factor of ∼0.5 compared with that at the end of cathode dark space [9], hence:
  (8/b) $$n_{e}\approx 1\times 10^{13}{\text{cm}}^{-3}$$

In an ELCAD-type discharge, the radial distribution of the *n_e_* electron density was determined from the measured Stark-broadening of the H_β_-486.1 nm line. In the negative glow:

(9) $$n_{e}=(8.5\pm 1.9)\times 10^{14}{\text{cm}}^{-3}$$

and in the positive column:

(10) $$n_{e}=(2.5\pm 0.5)\times 10^{14}{\text{cm}}^{-3}$$

was obtained [16], but in contrast with the *n_e_*(*r*) distribution for the positive column, the *n_e_* distribution for the negative glow (0.2 mm above the cathode!) exhibits an unbelievably constant and noiseless value in this publication. Such an extraordinary statistical parameter immediately generates the assumption of an instrumental error source instead of true measurement data. Perhaps the plasma position was inadequate and the naturally noisy negative glow was out of the observation line, and most probably the mirrored anode glow was observed in fact. Hence, the n_e_ value for the negative glow given by Equation (9) is erroneous.

**Figure 3.**
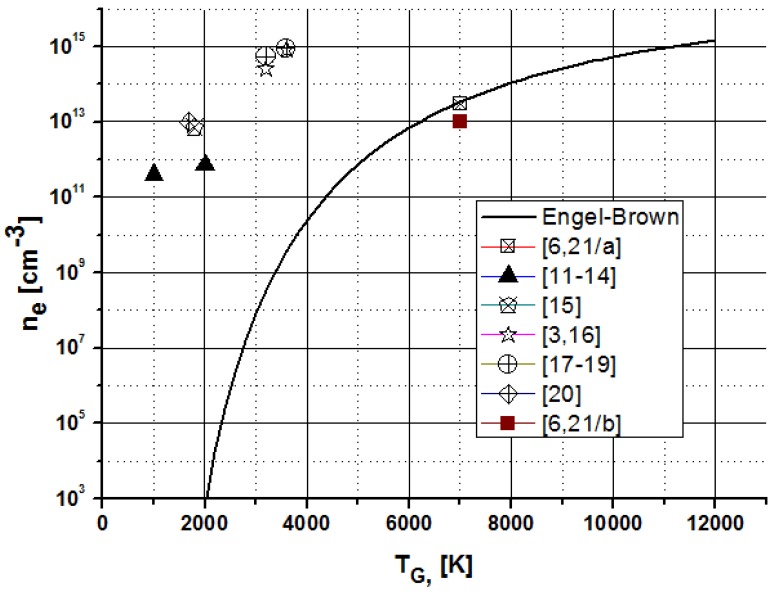
Published n_e_ and T_G_ data pairs for ELCAD and its homolog discharge plasmas. Solid curve represents the equilibrium parameters calculated for H_2_O vapor by the von Engel-S.C.Brown approximation (7). [6,21/a] and [6,21/b] points are due to the data of Equations (8/a) and (8/b).

Applying Equation (7), the evaluation of the published T_G_ and *n_e_* values presented in Table 1 can be summarized by a combined plot shown by Figure 3.

Figure 3 shows that the use of the N_2_ emission for investigation of the plasma core is misleading due to the fact that the plasma core in the negative glow does not contain components of the ambient atmosphere.

Except the T_G_ ≈ 7,000 K and *n_e_* ≈ (1–3) × 10^13^ cm^−3^ values [6,21], the published n_e_ electron density and the T_G_ gas temperature data pairs are very far from the von Engel equilibrium curve (solid line) calculated for H_2_O vapor. In accordance with the usual, classical readings, the error of temperatures presented on Figure 3, is about ∼2,500–9,000 K. On the other hand, the published electron density values are higher with about two orders of magnitude compared with the expected one.

## 4. Conclusions

The evaluation of the published data performed by means of Equation (3) shows that in most of the cases, the obtained T_rot_ rotational temperature and n_e_ electron density values are not consistent with each other. Generally, the obtained T_rot_ values are much lower, while the determined *n_e_* values are very much higher compared with those can be received from Equation (7). The main reasons of this are the following:

1. It is not yet widely understood that ELCAD plasmas operate in saturated H_2_O vapor due to the very intense sputtering of the aqueous cathode. Hence, the gas temperature determination based on the emitted spectrum of N_2_ molecule being only in the outer sheath cannot give the correct, real gas temperature data of the plasma.
2. For determination of T_G_ (≈T_rot_) in the ELCAD technique, the method of de Izarra giving T_rot_ from the ratio of the measured unresolved ultraviolet band heads of OH proved to be the only reliable method. These T_rot_ values were verified by an independent, interferometric measurement [33,34].
3. A serious conceptual confusion can be found in the interpretation of the temperature results. The T_e_ ≫ T_rot_ values obtained from the emitted spectrum simulation and the Boltzmann plot method were attributed to the fact that the investigated ELCAD plasma is not in a local thermodynamic equilibrium. But this is a self-contradiction, since the Maxwell-Boltzmann distribution was applied for determination of the temperatures. These could be avoided if T_G_ = T_e_ are used for the mutual validation of the received temperature data. A thorough evaluation of the experiments and the simulations calculations could help to find the possible error sources causing these incorrect temperature values.
4. The experimental determinations of the *n_e_* electron density refer to that the limit of the applied experimental methods were not presented, since often they were not taken into account. In certain cases, the theoretical determination of *n_e_* is too complicated and confusing, moreover it neglects the basic properties of the ELCAD plasma.
5. Some investigations of this “exotic” plasma seem to be loaded with serious experimental errors:
  - ambiguous plasma conditions (e.g., distilled water cathode without recording of the steeply changing pH and conductivity values of the cathode solution during the plasma operation)
  - plasma probing with low spatial resolution technique (e.g., the received values cannot be linked to the relevant parts of this plasma of small physical dimensions, V ∼ 10 mm^3^)
  - considering the small physical dimensions of the plasma, a misaligned optical system can easily produce meaningless results.

Generally in the reviewed publications the prime rule of the experimental research is frequently missing: the experiment must be as precise as the theoretical base of the evaluation, in other words the quality of the measured data determines the reliability of the evaluation results. Without a strictly designed experimental setup using adequate techniques fitting to the characteristics of the specimen to be investigated, only conclusions of low validity can be derived, even with applying the most sophisticated theoretical treatments.

## Figures and Tables

**Table 1.** The published T_rot_ ≈ T_G_ rotational (gas), T_e_ electron temperature and n_e_ electron density values showing the measuring place (NG = negative glow, PC = positive column, NAR = near anode region), the determination method, the type of discharge and the references.

**T_rot_ ≈ T_G_ [K]**	**T_e_ [K]**	**n_e_ [cm^−3^]**	**type of discharge**	**Ref.**
**NAR: 6,000** **PC: 4,800** **NG: 7,000** OH emission, Izarra method	**NAR: 6,000** **PC: 5,500** **NG: 7,000** Int. ratio of 510.5, 515.3 nm Cu lines	**NG: 10^13^; 3 × 10^13^** Calculated from the operating parameters	Original ELCAD	[6,21]
**NAR, PC: 1,000** **NG: 2,000** N_2_ emission	**4,000** Intensity ratio of H-Balmer lines	**PC: 4 × 10^11^** **NG: 7 × 10^11^** Microwave absorption	liquid electrodes	[11–14]
**1,800** N_2_ emission		**7 × 10^12^** Calculated from current density, temperature	a.c. excited (*v* = 60 Hz) ELCAD like	[15]
**PC: 3,200** **NG: 3,600** OH emission Boltzmann plot	**PC: 2,500** **NG: 5,000** from Fe-I lines, Boltzmann-plot; Saha-equation	**PC: 2.5 × 10^14^** **NG: 8.5 × 10^14^** Stark broadening of H-486.1 nm line	ELCAD type	[3,16]
**PC: 3,250** **NG: 3,400–3,600** OH emission **PC: 3,250** **NG: 2,200–2,800** N_2_ emission	**6,100** Intensity ratio of H_α_ and H_β_ lines	**NG:(5.5–9) × 10^14^** Stark broadening of H-486.1 nm	ELCAD like	[17–19]
**1,700** N_2_ emission **2,200–3,200** OH emission	**5,565** calculated from OH vibr. level population	**10^13^** calculated from current distribution	ELCAD like with distilled water cathode	[20]
